# Supplementary material for: Explainable Machine Learning with Pairwise Interactions for Predicting Conversion from Mild Cognitive Impairment to Alzheimer’s Disease Utilizing Multi-Modalities Data
Source: Brain Sci. 2023 Oct 31;13(11):1535. doi: 10.3390/brainsci13111535 (PMC10670176; doi:10.3390/brainsci13111535)
Supplement: Supplementary file 1 [file brainsci-13-01535-s001.zip › brainsci-2582247-supplementary.pdf]

Supplementary

For a more detailed visualization of the results of local explanation of EBM model, consider **Supplementary Figure S1**

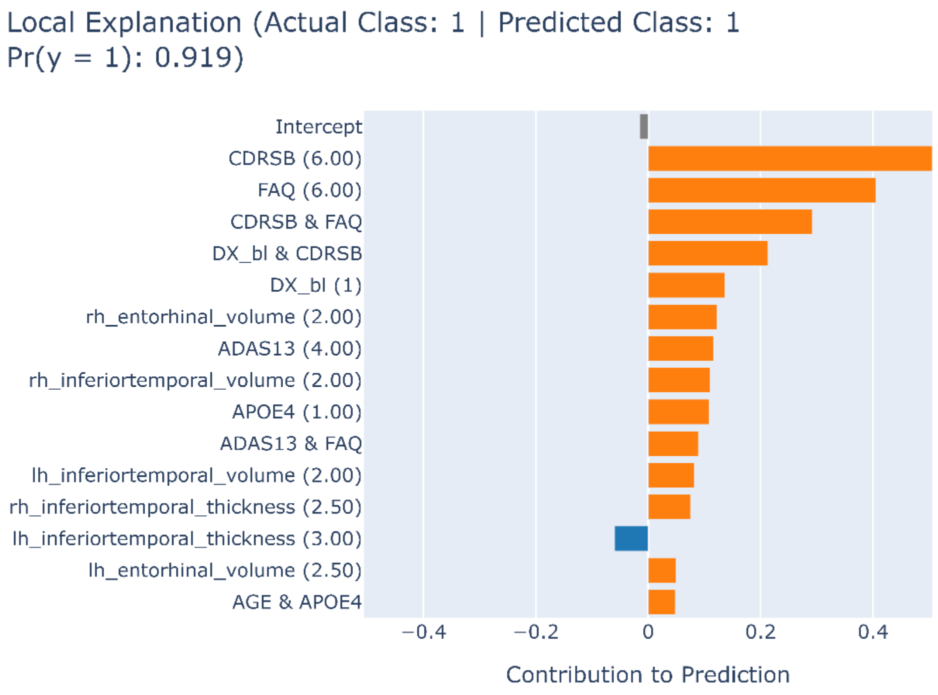

Supplementary Figure S1. The local interpretation provided by EBM model of Patient 1.

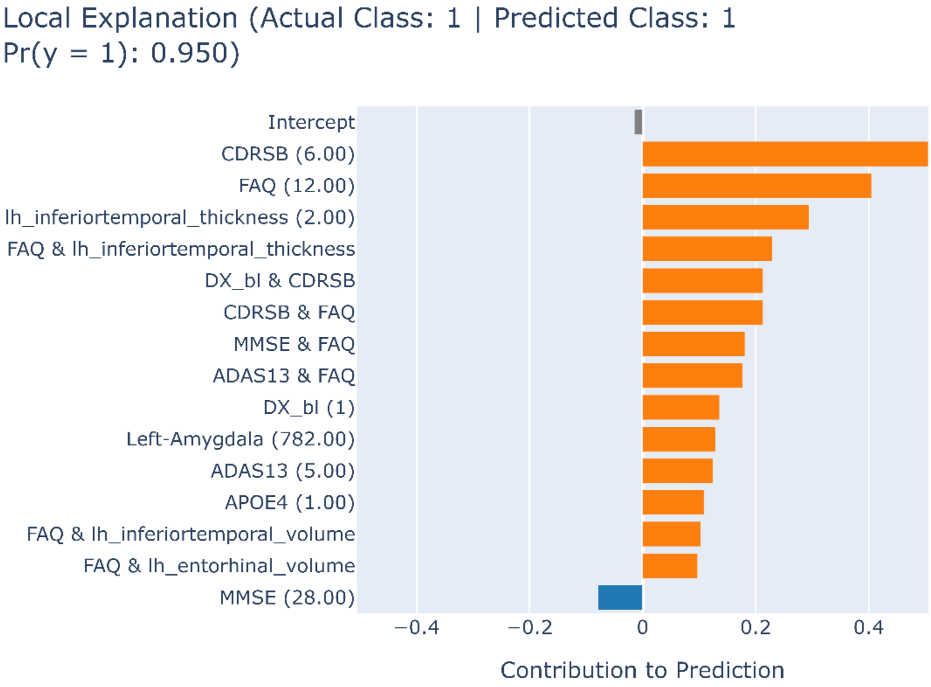

Supplementary Figure S2. The local interpretation provided by EBM model of Patient 2.

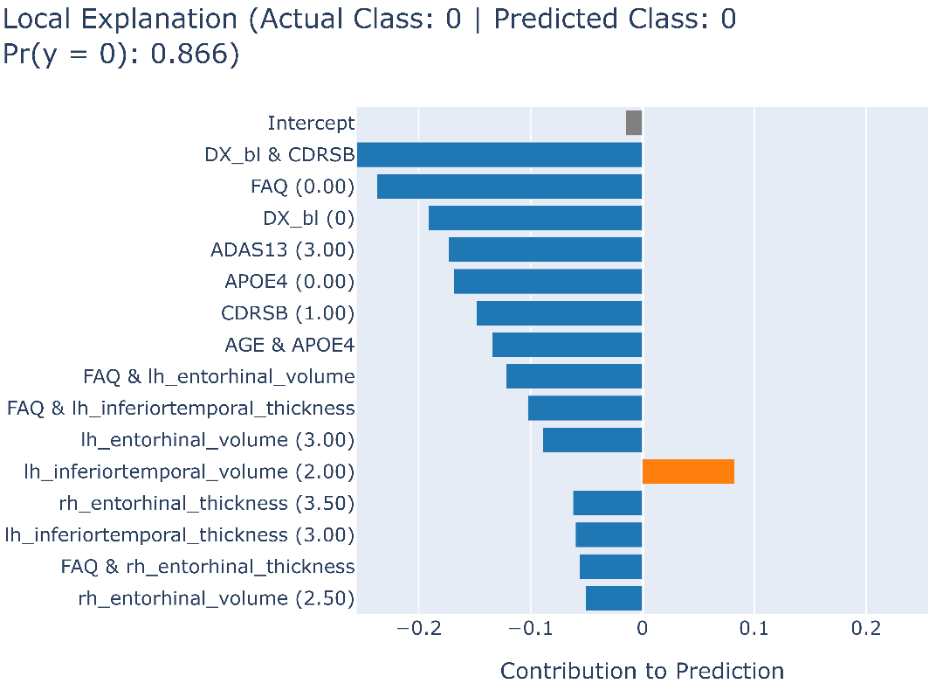

Supplementary Figure S3. The local interpretation provided by EBM model of Patient 3.

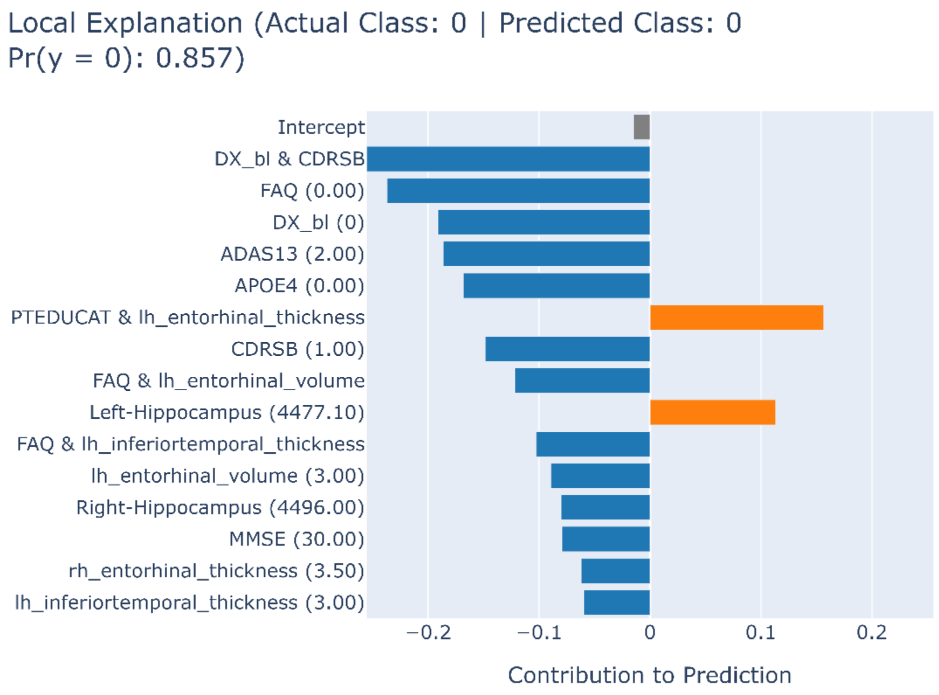

Supplementary Figure S4. The local interpretation provided by EBM model of Patient 4.
